# A systematic review of care management interventions targeting multimorbidity and high care utilization

**DOI:** 10.1186/s12913-018-2881-8

**Published:** 2018-01-30

**Authors:** Jennifer M. Baker, Richard W. Grant, Anjali Gopalan

**Affiliations:** Kaiser Permanente Northern California Division of Research, 2000 Broadway, Oakland, CA 94612 USA

**Keywords:** Utilization, Multimorbidity, Care management, Case management, Complex patients, Chronic condition, Depression

## Abstract

**Background:**

Evidence supporting the effectiveness of care management programs for complex patients has been inconclusive. However, past reviews have not focused on complexity primarily defined by multimorbidity and healthcare utilization. We conducted a systematic review of care management interventions targeting the following three patient groups: adults with two or more chronic medical conditions, adults with at least one chronic medical condition and concurrent depression, and adults identified based solely on high past or predicted healthcare utilization.

**Methods:**

Eligible studies were identified from PubMed, published between 06/01/2005 and 05/31/2015, and reported findings from a randomized intervention that tested a comprehensive, care management intervention. Identified interventions were grouped based on the three “complex” categories of interest (described above). Two investigators extracted data using a structured abstraction form and assessed RCT quality.

**Results:**

We screened 989 article titles for eligibility from which 847 were excluded. After reviewing the remaining 142 abstracts, 83 articles were excluded. We reviewed the full-text of 59 full-text articles and identified 15 unique RCTs for the final analysis. Of these 15 studies, two focused on patients with two or more chronic medical conditions, seven on patients with at least one chronic medical condition and depression, and six on patients with high past or predicted healthcare utilization. Measured outcomes included utilization, chronic disease measures, and patient-reported outcomes. The seven studies targeting patients with at least one chronic medical condition and depression demonstrated significant improvement in depression symptoms (ranging from 9.2 to 48.7% improvement). Of the six studies that focused on high utilizers, two showed small reductions in utilization. The quality of the research methodology in most of the studies (12/15) was rated fair or poor.

**Conclusions:**

Interventions were more likely to be successful when patients were selected based on having at least one chronic medical condition and concurrent depression, and when patient-reported outcomes were assessed. Future research should focus on the role of mental health in complex care management, finding better methods for identifying patients who would benefit most from care management, and determining which intervention components are needed for which patients.

**Electronic supplementary material:**

The online version of this article (10.1186/s12913-018-2881-8) contains supplementary material, which is available to authorized users.

## Background

Building on the success of single disease-focused care management interventions (e.g., diabetes, congestive heart failure), care management programs are now being implemented to manage the care of more complex patients [1, 2]. However, the definition of patient complexity is itself complex, with many definitions based on a myriad of different factors, ranging from functional limitations, to providers perceptions, to the presence of exacerbating factors (e.g., medical or mental illness, socioeconomic challenges, recent hospitalization, etc.) [3–5]. Several large reviews have attempted to synthesize the evidence regarding the efficacy of these programs across a wide range of patient complexity definitions, yet the findings have been inconclusive [1, 2]. One of the largest studies to date included 15 separate interventions implemented through the Medicare Coordinated Care Demonstration Project [1]. Unfortunately, despite the large project scale that included 18,402 participants, inferences from this study are limited by the variation among the 15 study sites in program design and intervention components. Moreover, overall success, even variously defined and measured, was modest. A systematic literature review conducted in 2013 by the Agency for Healthcare Research and Quality (AHRQ) also found that care management had limited impact on the quality of care and healthcare utilization for many of the examined types of complex patients [6]. Notably, neither of these completed reviews focused specifically on complex patients defined primarily by multimorbidity and high healthcare utilization.

Multimorbidity is increasing in America and is a major driver of high healthcare costs and spending. While half of all Americans have at least one chronic condition, one in four have two or more chronic medical conditions [7]. Beyond medical comorbidities, patients with even one chronic medical condition, like diabetes and heart disease, are disproportionately affected by comorbid depression [8, 9]. Multimorbid, complex patients consume a disproportionately large proportion of U.S. healthcare spending. Based on the 2010 Medical Expenditure Panel Survey data, 1% of the U.S. population accounted for 22% of healthcare costs, and 5% accounted for 50% of costs [10]. Motivated by these population trends, we conducted a focused systematic literature review of randomized clinical trials (RCTs) published in the past 10 years that implemented and described the effectiveness of outpatient care management interventions in three, specific types of complex patients: 1) Adults with two or more chronic medical conditions (the two aforementioned reviews only required only one or more chronic conditions to be included), 2) adults with at least one chronic medical condition and concurrent depression, and 3) high healthcare utilizers. Beyond assessing the effectiveness of these interventions, this review also sought to answer the following question: *What are the necessary components and appropriate intensity of effective care management interventions?*

## Methods

### Data sources and searches

We followed the Preferred Reporting Items for Systematic Reviews and Meta-Analysis (PRISMA) guidelines for systematic literature reviews (the PRISMA checklist is included in Additional file 1) [11]. This review was not registered with the International Prospective Register of Systematic Reviews (PROSPERO) as the study did not meet inclusion criteria for registration (key data extraction had already occurred). We electronically queried the U.S. National Library of Medicine National Institutes of Health (PubMed) for RCT studies using a pre-defined list of search terms with a combination of words relating to care management (e.g., “care management”; “case management”; “collaborative care”). A full list of search terms is available in Additional file 2: Table S1. We tracked and saved results of each search term and removed duplicate records. Records from sources other than PubMed were included by conducting backward and forward citation searches of identified articles (i.e., review of references in identified, eligible articles) and a search in ClinicalTrials.gov for ongoing, eligible RCTs.

### Study selection

We included studies meeting the following criteria: 1) RCT published between 06/01/2005 and 05/31/2015 (this 10 year window was chosen to ensure that the study findings would have present-day relevance); 2) tested a patient-focused, comprehensive care management intervention (areas of focus included some combination of self-management, healthcare system navigation, self-efficacy, symptom monitoring, symptom management, etc.) targeting the “whole” patient (e.g. including nurse- or case-manager led interventions, integrated care team strategies, group interventions); 3) intervention participants were 19 years or older based on Pubmed’s definition of adult age (children were excluded given their different care needs, comorbid diagnoses and types of interventions used compared to adults); 4) intervention participants belonged to one of the three complex categories of interest: a) two or more chronic medical conditions, b) at least one chronic medical condition + depression, and c) high past or predicted utilization – identified via past level of healthcare utilization and/or algorithms designed to predict future healthcare utilization; 5) assessed outcomes focused on measures of clinical quality, care processes, disease outcomes and/or measures of healthcare utilization (e.g., admissions, readmissions, costs); and, 6) the study was conducted in U.S., U.K., or other economically developed country based on Gross Domestic Product (GDP) with results published in English.

Two reviewers (JMB and AG) independently assessed all record titles for potential inclusion into the analysis, and a third reviewer (RWG) adjudicated when discrepancies arose. We excluded studies with titles that implied ineligibility (e.g., pre/post analysis). We then reviewed abstracts of the remaining records to remove studies that did not meet inclusion criteria. Full-text articles of remaining records were obtained for final screening using a structured abstraction form to collect key data elements from each study. The reviewers conducted a double-screening of included articles through data extraction and critical appraisal.

### Data extraction and quality assessment

Two reviewers (JMB and AG) each extracted the following data: patient eligibility criteria, selection and recruitment methods, care model, number of participants recruited, duration of intervention, mode of intervention, details of intervention components, number of contacts with patient during intervention, communication with a primary care physician (PCP), outcomes measured, results of outcomes measured, location of study, and funding source.

The same reviewers assessed the quality of the included studies using the National Institutes of Health (NIH) framework for the Quality Assessment of Controlled Intervention Studies [12]. This assessment protocol provides detailed guidelines for rating RCTs as Good, Fair, or Poor based on 14 objective measures of design and study implementation (e.g., method of randomization, retention rates, adherence to intervention protocols, and sample size). The third reviewer adjudicated when discrepancies arose.

### Data synthesis and analysis

Once full-text articles were identified for inclusion and data extraction was completed, the same two reviewers conducted a qualitative, iterative analysis. We aimed to determine patterns and associations between how complex patients were defined and identified; the mode, duration, and components of interventions; and types of outcomes measured and what those results showed.

As an exploratory analysis, we attempted to quantify the effect size of the measured depression outcomes by comparing the percent improvement in mean depression scores derived from the studies that measured depression symptoms. We calculated the percent improvement in mean depression scores post-intervention as the ratio of the difference in mean depression scores between the intervention and control groups over the control group’s mean depression score. We used the same approach for calculating the upper and lower 95% confidence intervals (CI) for the percent improvement in mean based on the reported confidence intervals. When the 95% CI were not reported, we calculated the standard error using the reported sample size, standard deviation, and/or *p*-value and assumed a normal distribution to construct them. Because studies used different scales to measure depression outcomes, percent improvement in mean depression scores was assessed. We were unable to apply this approach to other outcome measures because of the heterogeneity of measure definitions and relatively small number of similar measures assessed across studies.

## Results

**Fig. 1:**
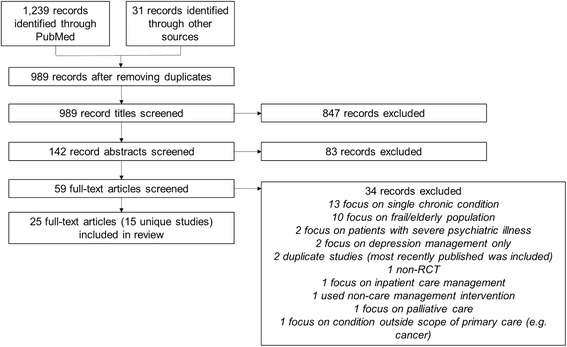
PRISMA flow diagram showing the sequential steps to reach the final number of included records for analysis

We screened 989 article titles for study eligibility, resulting in the exclusion of 847 articles. We reviewed the remaining 142 abstracts and excluded 83 additional articles. We reviewed the full-text of the remaining 59 full-text articles, thereby excluding 34 more articles. Fifteen unique RCTs were included for final analysis which included 25 full-text articles (some studies were reported in multiple articles; see Fig. 1) [1, 13–35]. A list of exclusion reasons can be found in Fig. 1. Eleven studies were conducted in the U.S., one in the U.K., one in Hong Kong, one in Sweden, and one in Australia.

The significant heterogeneity in the duration, intensity, and content of the reported interventions limited our ability to draw conclusions on the differential impact of intervention design on the examined outcomes. For most studies, the quality of the research methodology was rated as fair or poor quality: three studies (20%) were rated “Good” [16, 29, 35]; 10 studies (67%) were rated “Fair” [13, 14, 23–27, 31–33] and two studies (13%) were rated “Poor” [30, 34]. The most common reasons for a study being rated as “Fair” was small sample size and limited details on adherence to intervention protocols. The two studies rated as “Poor” had large differences in non-participation/drop-out rates between study arms, considered a “fatal flaw” by the assessment guidelines.

Of the 15 RCTs included in our review, two focused on patients with two or more chronic medical conditions, seven focused on patients with at least one chronic medical condition and concurrent depression, and six on patients with high past or predicted healthcare utilization. Of the two studies focused on patients with two or more chronic medical conditions, one included patients with heart disease and diabetes while the other used PCP-confirmed chronic multimorbidity. Of the seven chronic disease plus depression-focused studies, the primary medical co-diagnoses were diabetes and/or coronary heart disease (three studies), diabetes (three studies), and hypertension (one study). The six utilization-based studies were mixed in how patients were identified: three studies identified patients based on their prior utilization; two used models to predict future utilization; and one combined past and predicted future utilization. It was not possible to quantitate the overlap between participants identified by the three types of complexity. For example, it is possible that participants in studies targeting patients with two or more chronic medical conditions also had high past utilization.

The mode, duration, and number of contacts between patient and care managers varied across all studies (Table 1). Program duration ranged from one to 36 months, number of participants ranged from 64 to 2289, and frequency of contacts between patient and care manager ranged from 0.33 to 2.7 times per month. Program care managers communicated with participants face-to-face (12/15) and/or by phone (13/15), and most care managers were able to communicate with the participant’s PCP (12/15).

**Table 1:** Characteristics of Interventions

	Author, year [reference]	Location	Study #	N	Length (months)	Intensity (contacts/ month)	Care manager	In-person	Phone	Mail	Communicate w/ PCP
≥2 chronic medical conditions	Dunbar 2014 [34]	USA	1	71	1	4	Nurse	**✓**	**✓**		
	Chow 2014 [35]	HKG	2	281	1	4	Nurse	**✓**	**✓**		
≥1 chronic medical condition + depression	Bogner 2008 [13]	USA	3	64	1	5	Other	**✓** ^**a**^	**✓**		**✓**
	Ell 2010 [14, 15, 48]	USA	4	387	12	0.73	Social Worker		**✓**		**✓**
	Katon 2010 [16–22]	USA	5	214	12	1.73	Nurse	**✓**	**✓**		**✓**
	Coventry 2015 [23]	UK	6	387	3	1.5	Other	**✓**			
	Bogner 2010 [24]	USA	7	58	1	5	Other	**✓**	**✓**		**✓**
	Morgan 2013 [25]	AUS	8	400	12	0.33	Nurse	**✓**			**✓**
	Bogner 2012 [22]	USA	9	180	3	1.6	Other	**✓**	**✓**		**✓**
Past or predicted high utilization	Shannon 2006 [27]	USA	10	823	12	1	Other		**✓**	**✓**	**✓**
	Boult 2011 [28, 29]	USA	11	850	20	1	Nurse	**✓**	**✓**		**✓**
	Reinius 2013 [30]	SWE	12	268	12	2.7	Nurse		**✓**		**✓**
	MCCD ^b^ Washington [1]	USA	13	2289	36	1.2	Nurse	**✓**	**✓**		**✓**
	MCCD ^b^ CenVaNet [1]	USA	14	1445	36	1.4	Nurses & Social Workers	**✓**	**✓**		**✓**
	Sledge 2006 [33]	USA	15	96	12	1	Nurse	**✓**	**✓**		**✓**

^a^**✓**indicates the study included this intervention component

^b^ Medicare Coordinated Care Demonstration

Intervention programs applied a variety of care management strategies. Most programs included problem-solving, coping, self-management, and self-efficacy skills (11/15); referral and care navigation assistance (11/15); patient education (11/15); and/or symptom monitoring (10/15). Managing stigma was less common (3/15) and was only used in programs where depression was a focus. An overview of care management strategies used within each study can be found in Additional file 2: Table S2. In many cases, information on how these strategies were implemented was limited. For example, studies that provided patient education did not describe the educational materials used or how they were delivered (e.g., via brochure, book, or computer), making direct comparisons between studies’ care management approaches impossible.

The types of outcomes tracked and measured fell into three broad categories that mirrored system-level, clinician-level, and patient-level outcomes: 1) utilization (e.g., emergency department visits, hospitalizations, outpatient visits, costs, and mortality); 2) clinical measures related to chronic conditions (e.g., hemoglobin A1c, lipids, blood pressure, body mass index, depression score, and medication adherence); and 3) patient-reported outcomes (e.g., patient satisfaction, health-related quality of life, self-efficacy, self-management, adherence to care plan, lifestyle changes, and quality of care). Of the seven studies targeting patients with at least one chronic medical condition and concurrent depression, all seven demonstrated significant improvement in depression symptoms. Moreover, 5/7 also showed improvement in at least one chronic medical condition-related outcome (Table 2). Overall, only seven of the 15 studies (47%) measured at least one healthcare utilization outcome, and six of these seven studies targeted patients identified by utilization patterns. Of these six studies focused on high utilizers, two showed a statistically significant change in one or more utilization measures that favored the intervention (Table 2). The two studies targeting patients with two or more chronic medical conditions primarily assessed patient-reported outcomes, and neither included any chronic condition-specific outcomes. Patient-reported outcomes were commonly assessed across all three complexity categories, and 8/15 (53%) reported significant improvement in at least one of these measures. Of the five studies measuring patient satisfaction, four reported a significant increase. A summary of patient-reported outcomes can be found in Additional file 2: Table S3. Although there were small numbers of studies with common outcome measures, interventions appeared more likely to be successful when: 1) participants were selected based on having at least one chronic medical condition ***and*** concurrent depression, and 2) patient-reported measures were included in the assessed outcomes.

**Table 2:** Utilization and Chronic Condition Measures and Outcomes

Utilization Measures							Chronic disease-related measures					
	Study #	ED visits	Hospital readmission	Outpatient physician visits	Cost	Mortality	HbA1C	Lipids	Blood pressure	BMI	Depression symptoms	Med adherence
≥2 chronic medical conditions	1											
	2		✓↓*									
≥1 chronic medical condition + depression	3								✓↓*		✓↓*	✓↑*
	4						**✓**			**✓**	✓↓*	
	5				✓↓*		✓↓*	✓↓*	✓↓*		✓↓*	✓↑*
	6										✓↓*	
	7						✓↓*				✓↓*	✓↑*
	8						**✓**	**✓**	**✓**	**✓**	✓↓*	
	9						✓↓*				✓↓*	✓↑*
Past or predicted high utilization	10	**✓**	✓↓*	✓↑*								
	11	**✓**	**✓**	**✓**								
	12	**✓**	**✓**	✓↓*	✓↓*	**✓**						
	13		**✓**		✓↑*							
	14		**✓**		✓↑*							
	15	**✓**	**✓**	✓↓*	**✓**							
Total # measuring outcome		4	7	4	5	1	4	2	3	2	7	4
Total # with significant change favoring the intervention		0	2	1	2	0	3	1	2	0	7	4

✓ Indicates the study measured this outcome

↑* Indicates an increasing trend among intervention group with statistical significance at *p* ≤ 0.05

↓* Indicates a decreasing trend among intervention group with statistical significance at *p* ≤ 0.05

**Fig. 2:**
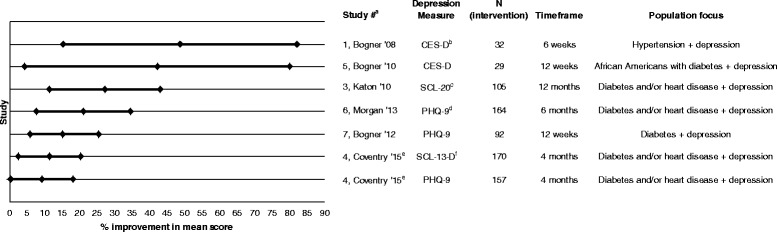
A summary of the effect size of depression outcomes among studies where depression was a criterion for intervention selection

While all the included studies demonstrated a benefit on at least one measured outcome, there was heterogeneity in how measures were defined and results reported. All seven of the studies focused on at least one chronic medical condition with concurrent depression showed a statistically significant improvement in depression symptoms; however, these differences were assessed and reported differently. Reported differences favoring the intervention ranged from a 9.9 to 19.3 point (*p* < 0.01) difference in Center for Epidemiologic Studies Depression Scale (CES-D) scores [13, 36], to 62% to 44% (*p* < 0.001) of participants with ≥50% reduction in depression symptoms [14], to 58.7% to 30.7% (*p* < 0.001) of participants with a Patient Health Questionnaire-9 (PHQ-9) score less than five at follow-up [26, 37]. However, we did compare effect size of depression outcomes across these studies. A summary of the effect size of depression outcomes among these studies can be seen in Fig. 2. Effect sizes ranged from a 9.2 to 48.7% improvement in mean depression scores (assessed as percent change to account for differences in scales). We were unable to include one study [14] in this analysis because the mean depression scores were not reported.

Among this same group of seven studies, the reporting of chronic medical disease-related outcomes also varied. For example, between groups, hemoglobin A1c (HbA1C) differences favoring care management included a difference of 6.7% vs 7.9% (*p* < 0.05) in mean HbA1C values [24], a difference of 0.58% change in HbA1C (*p* < 0.001) between intervention arms [16], and a 60.9% vs 35.7% (*p* < 0.001) difference in the proportion achieving an HbA1C ≤7% at follow-up [26]. Differences in blood pressure were reported as either a difference in mean blood pressure or as a change in blood pressure, the largest significant finding favoring care management was a difference in mean systolic blood pressure of 127.3 mmHg to 141.3 mmHg (*p* < 0.001) [13]. Additionally, within the seven studies, two did not show significant improvement on other measures other than self-reported depression outcomes [4, 8]. These two studies had less < 1 contacts/month while the other studies had ≥1 contact/month [3, 5, 7, 9].

Utilization outcomes were also measured and reported in a variety of ways. Seven studies measured hospital admissions and readmissions in the post-intervention period; however, only two studies reported significant reductions in hospital readmissions. The first study measured hospital readmission in two timeframes (28 days and 84 days post-hospital discharge). The 28-day readmission outcome rate showed no significant change between cases and controls (23% vs. 15%, *p* = 0.311), while the 84-day readmission showed a significant reduction between study arms (45% vs. 33%, *p* = 0.018) [35]. The second study reported the intervention arm was 57% less likely than controls to have increased hospital admissions in the 12-months post-intervention (OR 0.43, 95% CI 0.22 – 0.84, *p* < 0.01) [27]. Five studies measured the cost-effectiveness of interventions with two reporting significant reductions in cost. Within these two studies, one reported a $594 per patient cost reduction in outpatient care in the 12 months post-intervention, but reported inconclusive results regarding inpatient costs [20]. The second study reported a 45% reduction (− 11,878€/patient [~$13,400/patient], *p* = 0.004) in total patient costs (planned and emergency care) in the 12 months post-intervention [30].

The clinical meaningfulness of the observed significant changes in patient-reported outcomes is difficult to quantify; as with other the clinical and utilization outcomes, different scales and statistical methods were used to report on these findings. Furthermore, limited information was available to assess how such differences translated to differences in related clinical and utilization measures.

## Discussion

In this review of RCTs testing care management interventions, we focused on three, increasingly common, types of patient complexity: 1) Two or more chronic medical conditions 2) at least one chronic medical condition with concurrent depression, and 3) high past or predicted utilization. We found only two studies that focused on patients with two or more chronic medical conditions; the seven studies focusing on patients with at least one chronic medical condition and depression were most likely to have positive results. Few of the six studies targeting high healthcare utilizers demonstrated a reduction in utilization measures. Of note, the large majority of studies were of fair or poor quality.

We identified only two studies that specifically focused on patients with two of more chronic medical conditions. The paucity of research focused specifically on multimorbidity is concerning given current U.S. multimorbidity prevalence and the associated costs. Americans experience a high prevalence of chronic diseases [7], and healthcare spending for adults with multimorbidity is seven-fold higher than individuals with only one chronic condition [38]. Indeed, multimorbidity has been called the most “common chronic condition” in health care [39]. A notable limitation of these two studies was a focus on patient-reported outcomes rather than chronic medical condition-related outcomes or utilization metrics.

Studies that enrolled patients with at least one chronic medical condition complicated by depression tended to improve both depression and chronic condition-related outcomes, specifically blood pressure or HbA1C. One explanation for the observed benefits is the tailoring of the intervention to address common barriers faced by patients with depression and chronic medical disease, specifically medication adherence and managing stigma. Another explanation may be that for care management to effectively improve chronic medical condition outcomes, the intervention must also address mental health conditions (e.g., depression) that may be co-occurring. This would not be unexpected given the well-documented overlap between chronic conditions like diabetes and heart disease with depression [8, 9, 40, 41].

We found few examples where care management meaningfully improved utilization outcomes. All the studies that identified complex patients based on their healthcare utilization included hospital readmissions as a primary outcome of interest. However, only two of these studies showed an improvement in this metric. This inconclusive finding is consistent with prior research demonstrating the complex relationship between the level of healthcare access and the likelihood of hospital readmission; both low and high levels of access to healthcare services have been associated with an increased likelihood of hospital readmissions [42–44]. Cost was another outcome of high interest in this group of studies. Only one of the included RCTs in this group noted a significant decrease in overall costs. Of note, this study employed a more rigorous method to identify patients for inclusion than any of the other included studies: after an initial screen for eligibility based on past utilization, a second qualitative screen was performed by two physicians to determine patients most likely to benefit from the care management [30]. A weakness of this high utilizer group of studies was the narrow scope of outcome measures. The outcomes examined were primarily utilization-related (e.g., cost, hospitalizations), and very few chronic medical disease-related outcomes and patient-reported outcomes were described, limiting a more comprehensive assessment of these care management interventions.

Among all of the included studies, common methodologic issues limited our ability to draw conclusions regarding the effectiveness of specific intervention components. For example, insufficient detail on implementation fidelity and participant adherence to the interventions limited any substantive observations on the relationships between intervention content and intensity and any patient benefits. Another common limitation across the three groups of studies was the marked heterogeneity of the examined outcomes. Included outcomes reflected the type of complex patient targeted (e.g., utilization outcomes in studies focused on high utilizers) or were too limited in their scope (e.g., only patient-reported outcomes). This restricted the ability to assess the differential impact of varying interventions and components on different outcomes in different types of complex patients.

Our results must be considered in the context of the study design. First, our analysis was primarily a qualitative review of the eligible studies because the heterogeneity of interventions, common methodologic shortcomings across many of the studies, and the small sample size of our review precluded a more rigorous quantitative comparison. However, we were able to quantify effect size in the subset of interventions measuring depression outcomes, and we demonstrated that, in aggregate, these studies showed a statistically significant improvement in depression scores. Second, we were only able to identify a limited number of studies, and the overall quality of the identified studies was fair. This likely reflects the challenges faced by researchers attempting to develop, implement, and rigorously evaluate multi-faceted interventions for complex patients in real-world settings. Third, by stratifying studies into three, very specific types of complexity, we further limited the number of studies in each category available for comparison. Our decision to stratify in this way reflected currently incomplete knowledge regarding the effectiveness of care management and the type of complexity being addressed. Fourth, we were only able to report on the outcomes as they were assessed in the identified studies. Given this, we can only acknowledge any limitations that may be present in these reported outcomes measures (e.g., different ways to measure medication adherence). Last, the review includes studies identified from a limited number of databases. While we conducted a thorough search within the included data sources, it is possible studies from other data sources that meet inclusion criteria were not included.

## Conclusions

To date, rigorously conducted clinical trials have not yet demonstrated a clear and clinically meaningful benefit of care management for complex patients. Given that many of the individual elements of care management (e.g., patient education, care team communication, care planning) have a clear benefit, the lack of compelling clinical trial evidence for integrated care strategies is disappointing. Our systematic review has underscored several areas where further research is needed. First, we need a greater understanding of the role of mental health in care management. The positive results seen in the seven studies targeting individuals with at least one chronic medical disease and concurrent  depression suggest that addressing depression may be a critical part of effective chronic condition care management. Still, the specific components driving these positive findings, along with their impact on patients’ long-term clinical outcomes and care utilization, remain unknown. Currently, the majority of primary care practices use care management significantly less often for treating depression than for other chronic illnesses [45]. This imbalance is concerning given recent evidence demonstrating that depression care management may improve mortality risk among complex patients three-fold compared to patients who do not receive depression care [46]. Second, we need better methods for identifying patients who would benefit most from care management. Simply sorting by disease category or employing data-driven algorithms may be inadequate; provider input and expertise may be required [47]. Finally, while interventions for complex patients tend to be multi-modal, further evidence is needed to determine which components of these interventions, and with what degree of intensity, is needed for which patients. One important step is the standardization of examined outcome measures (e.g., the inclusion of common chronic disease-related measures) to better enable direct comparisons between interventions. Advances in these areas will inform future efforts to identify and effectively tailor effective care programs for complex patients.

## Additional files

Additional file 1: PRISMA Checklist. (DOC 63 kb)

Additional file 2: Table S1. Search Terms. **Table S2.** Content of Interventions. **Table S3.** Patient-reported Measures and Outcomes. (DOCX 24 kb)

## Abbreviations

AHRQ: Agency for Healthcare Research and Quality
CES-D: Center for Epidemiologic Studies Depression Scale
HbA1C: Hemoglobin A1c
NIH: National Institutes of Health
PCP: Primary Care Provider
PHQ-9: Patient Health Questionnaire-9
RCT: Randomized Controlled Trial
