# Supplementary material for: A systematic review of care management interventions targeting multimorbidity and high care utilization
Source: BMC Health Serv Res. 2018 Jan 30;18:65. doi: 10.1186/s12913-018-2881-8 (PMC5791200; doi:10.1186/s12913-018-2881-8)
Supplement: Supplementary file 2 — Search Terms. Table S2. Content of Interventions. Table S3. Patient-reported Measures and Outcomes. (DOCX 24 kb) [file 12913_2018_2881_MOESM2_ESM.docx]

Additional file 1

| **Table S1: Search Terms** | |
| --- | --- |
| **Search term** | **# of results** |
| “care management” | 408 |
| “case management” | 405 |
| “collaborative care” | 183 |
| “chronic condition” and “care” | 43 |
| "Case management" or "care management" or "collaborative care" and "clinical trial" and "chronic" | 32 |
| "Case management" or "care management" or "collaborative care" and "chronic" | 166 |
| "care management" and "complex patients" | 1 |
| "case management" and "complex patients" | 1 |
|  |  |
| # of results after duplicates removed: | 958 |

| Table S2. Content of Interventions | | | | | | | | | |
| --- | --- | --- | --- | --- | --- | --- | --- | --- | --- |
|  | Study # | Patient education | Symptom monitoring | Referral /care navigation | Care plan/goals | Managing Stigma | Medication adherence | Problem solving/coping/self-management/self-efficacy | Lifestyle (diet, exercise, stress) |
| ≥2 chronic medical conditions | 1 | **✓** | **✓** |  |  |  | **✓** | **✓** | **✓** |
|  | 2 |  | **✓** |  | **✓** |  | **✓** | **✓** | **✓** |
| ≥1 chronic medical condition + depression | 3 | **✓** | **✓** | **✓** | **✓** | **✓** | **✓** |  |  |
|  | 4 |  | **✓** | **✓** |  | **✓** |  | **✓** |  |
|  | 5 | **✓** | **✓** |  | **✓** |  | **✓** | **✓** | **✓** |
|  | 6 | **✓** | **✓** | **✓** | **✓** |  | **✓** |  | **✓** |
|  | 7 | **✓** | **✓** | **✓** | **✓** | **✓** | **✓** |  |  |
|  | 8 | **✓** |  |  | **✓** |  |  | **✓** | **✓** |
|  | 9 | **✓** | **✓** | **✓** |  |  | **✓** | **✓** |  |
| Past or predicted high utilization | 10 |  |  | **✓** | **✓** |  |  |  |  |
|  | 11 | **✓** | **✓** | **✓** | **✓** |  |  | **✓** | **✓** |
|  | 12 | **✓** |  | **✓** | **✓** |  |  | **✓** |  |
|  | 13 | **✓** |  | **✓** | **✓** |  | **✓** | **✓** | **✓** |
|  | 14 | **✓** |  | **✓** | **✓** |  | **✓** | **✓** |  |
|  | 15 |  | **✓** | **✓** | **✓** |  |  | **✓** |  |
| Total # that included this component | | 11 | 10 | 11 | 12 | 3 | 9 | 11 | 7 |

✓: Indicates the study included this intervention component

| Table S3. Patient-reported Measures and Outcomes | | | | | | | | | |
| --- | --- | --- | --- | --- | --- | --- | --- | --- | --- |
|  | Study # | Patient satisfaction | Health-related Quality of life | Self-efficacy | Self-management skills | Adherence to care plan | Lifestyle changes (diet/exercise) | Quality of care | Self-rated health |
| ≥2 chronic medical conditions | 1 |  | **✓↑*** | **✓↑*** | **✓↑*** |  | **✓↑*** |  |  |
|  | 2 |  | **✓↑*** | **✓↑*** |  |  |  |  | **✓↑*** |
| ≥1 chronic medical condition + depression | 3 | **✓↑*** |  |  |  |  |  |  |  |
|  | 4 | **✓↑*** | **✓↑*** | **✓↑*** | **✓** |  |  |  |  |
|  | 5 | **✓↑*** | **✓** | **✓** |  | **✓↑*** | **✓** |  |  |
|  | 6 | **✓↑*** | **✓↑*** | **✓↑*** | **✓↑*** |  |  | **✓** |  |
|  | 7 |  |  |  |  |  |  |  |  |
|  | 8 |  |  |  |  |  | **✓** |  |  |
|  | 9 |  |  |  |  |  |  |  |  |
| Past or predicted high utilization | 10 |  |  |  |  |  |  |  |  |
|  | 11 |  |  |  |  |  |  | **✓↑*** |  |
|  | 12 |  | **✓** |  |  |  |  |  | **✓↑*** |
|  | 13 |  |  |  |  |  | **✓** | **✓** |  |
|  | 14 |  |  |  |  |  | **✓** | **✓** |  |
|  | 15 | **✓** | **✓** |  |  |  |  |  |  |
| Total # measuring outcome | | 5 | 7 | 5 | 3 | 1 | 5 | 4 | 2 |
| Total # with significant change in outcome favoring the intervention | | 4 | 4 | 4 | 2 | 1 | 1 | 1 | 2 |

✓: Indicates the study measured this outcome

↑*: Indicates an increasing trend among intervention group with statistical significance at p≤0.05↓*: Indicates a decreasing trend among intervention group with statistical significance at p≤0.05
